# The Expression and Prognostic Value of SUMO1-Activating Enzyme Subunit 1 and Its Potential Mechanism in Triple-Negative Breast Cancer

**DOI:** 10.3389/fcell.2021.729211

**Published:** 2021-09-21

**Authors:** Qingshui Wang, Wenting Zhong, Lin Deng, Qili Lin, Youyu Lin, Hongxia Liu, Luyun Xu, Lingfang Lu, Yajuan Chen, Jianping Huang, Meichen Jiang, Han Xiao, Jie Zhang, He Li, Yuxiang Lin, Chuangui Song, Yao Lin

**Affiliations:** ^1^Central Laboratory at the Second Affiliated Hospital of Fujian Traditional Chinese Medical University, Collaborative Innovation Center for Rehabilitation Technology, Fujian University of Traditional Chinese Medicine, Fuzhou, China; ^2^Key Laboratory of Optoelectronic Science and Technology for Medicine of Ministry of Education, College of Life Sciences, Fujian Normal University, Fuzhou, China; ^3^Fujian Provincial Key Laboratory of Hepatic Drug Research, Fuzhou, China; ^4^Department of General Surgery, The 900th Hospital of the Joint Logistics Support Force, Fuzhou, China; ^5^Department of Pathology, Fujian Medical University Union Hospital, Fuzhou, China; ^6^Department of Breast Surgery, Fujian Medical University Union Hospital, Fuzhou, China; ^7^Department of General Surgery, Fujian Medical University Union Hospital, Fuzhou, China; ^8^Breast Cancer Institute, Fujian Medical University, Fuzhou, China

**Keywords:** TNBC, SAE1, WGCNA, prognosis, cell cycle

## Abstract

**Background:** Triple-negative breast cancer (TNBC) is the most invasive and metastatic subtype of breast cancer. SUMO1-activating enzyme subunit 1 (SAE1), an E1-activating enzyme, is indispensable for protein SUMOylation. SAE1 has been found to be a relevant biomarker for progression and prognosis in several tumor types. However, the role of SAE1 in TNBC remains to be elucidated.

**Methods:** In the research, the mRNA expression of SAE1 was analyzed *via* the cancer genome atlas (TCGA) and gene expression omnibus (GEO) database. Cistrome DB Toolkit was used to predict which transcription factors (TFs) are most likely to increase SAE1 expression in TNBC. The correlation between the expression of SAE1 and the methylation of SAE1 or quantity of tumor-infiltrating immune cells was further invested. Single-cell analysis, using CancerSEA, was performed to query which functional states are associated with SAE1 in different cancers in breast cancer at the single-cell level. Next, weighted gene coexpression network (WGCNA) was applied to reveal the highly correlated genes and coexpression networks of SAE1 in TNBC patients, and a prognostic model containing SAE1 and correlated genes was constructed. Finally, we also examined SAE1 protein expression of 207 TNBC tissues using immunohistochemical (IHC) staining.

**Results:** The mRNA and protein expression of SAE1 were increased in TNBC tissues compared with adjacent normal tissues, and the protein expression of SAE1 was significantly associated with overall survival (OS) and disease-free survival (DFS). Correlation analyses revealed that SAE1 expression was positively correlated with forkhead box M1 (FOXM1) TFs and negatively correlated with SAE1 methylation site (cg14042711) level. WGCNA indicated that the genes coexpressed with SAE1 belonged to the green module containing 1,176 genes. Through pathway enrichment analysis of the module, 1,176 genes were found enriched in cell cycle and DNA repair. Single-cell analysis indicated that SAE1 and its coexpression genes were associated with cell cycle, DNA damage, DNA repair, and cell proliferation. Using the LASSO COX regression, a prognostic model including SAE1 and polo-like kinase 1 (PLK1) was built to accurately predict the likelihood of DFS in TNBC patients.

**Conclusion:** In conclusion, we comprehensively analyzed the mRNA and protein expression, prognosis, and interaction genes of SAE1 in TNBC and constructed a prognostic model including SAE1 and PLK1. These results might be important for better understanding of the role of SAE1 in TNBC. In addition, DNA methyltransferase and TFs inhibitor treatments targeting SAE1 might improve the survival of TNBC patients.

## Introduction

Triple-negative breast cancer (TNBC) is a specific type of breast cancer which lacks the expression of estrogen receptor (ER), progesterone receptor (PR), and human epidermal growth factor receptor (HER2) (Jhan and Andrechek, 2017; Vagia et al., 2020). Although TNBC only comprises approximately 15–20% of all breast cancers, it has a higher recurrence and mortality rate than other types of breast cancer (Sporikova et al., 2018; da Silva et al., 2020). Patients with TNBC generally develop distant metastasis within the first 3 years after initial treatment, with the mortality rate could reach about 40% in the first 5 years (Wilson et al., 2019; Chang-Qing et al., 2020). Generally, TNBC do not respond to hormone or HER2-targeted therapies due to the absence of ER, PR, and HER2. Therefore, a combination of surgery, chemotherapy, and radiotherapy remains the most common treatment strategy for TNBC patients. While significant advances have been made in the treatment of TNBC, the prognosis of these patients remains extremely poor. Therefore, it is still necessary to identify new predictive biomarkers for treatment response and promise therapeutic targets for TNBC.

SUMOylation is a key post-translational modification with the form of conjugation of SUMO molecule to an acceptor lysine of target proteins, which is catalyzed by E1-activating enzyme, E2-conjugating enzyme, and several E3-ligating enzymes (Hickey et al., 2012; Pichler et al., 2017; Wang Q. et al., 2019; Chang and Yeh, 2020). SUMO1-activating enzyme subunit 1 (SAE1), an E1-activating enzyme, is indispensable for protein SUMOylation. SAE1 has a molecular weight of 110 kDa, predominantly localizes to the nucleus and can be found in a variety of tissues. SAE1 forms a thioester bond between SUMO protein and UBA2/SAE2 by regulating the activation of ATP-dependent SUMO protein, and then participates in the process of SUMO protein modification (Amente et al., 2012; Yang et al., 2019; Wang et al., 2020; Ong et al., 2021). SAE1 mainly mediates acetylation and phosphorylation and plays an important role in chromosome division and apoptosis. It has been found that SUMOylation is an important mechanism in cellular responses to stress and one that is usually abnormal in many cancers (Song et al., 2014; Yang et al., 2019). For instance, SUMO E3 protein inhibitor of activated STAT3 (PIAS3) is upregulated in various cancer types, like prostate, lung, colon, and breast cancer and brain tumor (Abbas et al., 2015; Li et al., 2015; He et al., 2018; Polimeno et al., 2020; Tseng et al., 2020). Recently, research and function of SAE1 have been increasingly analyzed in organism. SAE1 has been found to play a significant role in various tumorigenesis and development, such as glioma, colon cancer, and non-small cell lung cancer (Yang et al., 2019; Zhang et al., 2019). Nevertheless, its functions of SAE1 in TNBC are indistinct by far. Whether SAE1 can be used as a therapeutic target for TNBC and its biological roles remain to be further studied.

## Materials and Methods

### Extraction of SUMO1-Activating Enzyme Subunit 1 Expression and Clinical Data From Triple-Negative Breast Cancer Datasets

The mRNA expression of SAE1 and clinical data for TNBC patients were extracted from the cancer genome atlas (TCGA)^1^ and gene expression omnibus (GEO) database^2^ including GSE21653 (Sabatier et al., 2011b), GSE31448 (Sabatier et al., 2011a), GSE45827 (Gruosso et al., 2016), GSE53752, GSE65216 (Maire et al., 2013), GSE36295 (Merdad et al., 2014), GSE37751 (Terunuma et al., 2014), GSE38959 (Komatsu et al., 2013), and GSE61724 (Mathe et al., 2015). The method for extracting microarray gene expression values is based on our previous research (Mathe et al., 2015; Wang Q. S. et al., 2019). First, the probe ID was converted into a gene symbol. When a gene was mapped to different probes, the genic expression value was calculated by the average expression value. Next, the median normalization was performed using the robust multichip averaging method. Kaplan-Meier survival curve was conducted to estimate the overall survival (OS) and disease-free survival (DFS) by using TCGA and GSE21653, respectively.

### Patients and Specimens

Two hundred seven patients from Fujian Medical University Union Hospital between August 2013 and October 2017 were retrospectively enrolled in this study. All 207 patients were histologically confirmed TNBC, with a median age of 55 years (range, 27–72 years). Relevant clinicopathological information, including tumor size, nodal status, tumor grade, lymphovascular invasion (LVI), type of surgery, chemotherapy, and radiotherapy status, were obtained from patients’ medical records. OS was defined as the time from the date of diagnosis until death from any cause. DFS was defined as the time from the date of diagnosis to the date of clinical relapse (with histopathology confirmation or radiological evidence of tumor recurrence). The last follow-up was December 31, 2020. All patients met the following inclusion criteria: (1) no history of other malignant tumors or bilateral breast cancer; (2) received post-operative adjuvant chemotherapy with at least three cycles of anthracycline-based and three cycles of taxane-based regimens; (3) primary tumor size was pT1c-pT2 (1 cm < *T* ≤ 5 cm); and (4) demographic, clinicopathological, and follow-up information were complete. This study was approved by the Research Ethics Committee of Fujian Medical University Union Hospital and informed consent was obtained from each participant.

### Immunohistochemistry Staining Analysis

We conducted immunohistochemistry (IHC) staining analysis to measure the protein expression of SAE1 in TNBC tissues and adjacent normal breast tissues according to the standard immunoperoxidase staining procedure. Slides were incubated with anti-SAE1 (ab97523, Abcam, Cambridge, United Kingdom, diluted 1:300) according to the manufacturer’s instructions. A negative control was also prepared *via* substitution of a primary antibody with phosphate-buffered saline (PBS). The IHC staining scores of SAE1 were evaluated by two independent pathologists. The percentage of stained positive cells was calculated from 1 to 4: 1, 0–25%; 2, 26–50%; 3, 51–75%; and 4, 75–100%. The staining intensity score was ranged from 0 to 3: 0, no staining; 1, weak staining; 2, moderate staining; and 3, strong staining. The percentage of positive tumor cells and the staining intensity were multiplied to produce a weighted score for each case. A score of 8–12 was defined as high expression level and a score of 0–7 was defined as low expression.

### Methylation Analysis

The methylation site and beta values of SAE1 promoter in TNBC was obtained from UCSC Xena Browser^3^. The beta values of CpG islands had been acquired using the Illumina HumanMethylation450K platform and had been preprocessed by TCGA by using standard protocols. One of the most widely used techniques to measure DNA methylation is the Illumina Infinium HumanMethylation450 BeadChip array, which covers approximately 450,000 CpG sites. At each CpG site, methylation is quantified by the beta value beta.

### Weighted Gene Correlation Network Analysis

Weighted gene coexpression network (WGCNA) is a common algorithm for building gene coexpression networks and performed through WGCNA R package (Langfelder and Horvath, 2008). The WGCNA hypotheses that the coexpression gene network follows the scale less distribution firstly, defines the adjoining function of the gene coexpression correlation matrix and gene network formation, calculates the difference coefficient of different nodes, and constructs a hierarchical cluster tree accordingly finally.

### Gene Ontology and Kyoto Encyclopedia of Genes and Genomes Pathway Enrichment Analysis

Gene Ontology (GO) analysis and kyoto encyclopedia of genes and genomes (KEGG) pathway enrichment were calculated by functional enrichment tool DAVID^4^ (Huang da et al., 2009). DAVID bioinformatics resources provide an integrated biological database and a repository of analytic tools for systematic exploration of biological meaning of gene set DAVID. The default parameters in the tool were used, and enriched pathways were ranked according to their enrichment scores. A *P*-value of <0.05 was identified as enriched functions.

### Single-Cell Analysis

CancerSEA^5^ (Yuan et al., 2019) depicts single-cell functional status maps that contain 14 functional states (including angiogenesis, apoptosis, invasion, EMT, differentiation, proliferation, DNA damage, metastasis, hypoxia, inflammation, cell cycle, DNA repair, stemness, and quiescence) from 25 types of cancer including 41,900 individual cells. CancerSEA was used to explore the potential roles of SAE1 and its coexpression genes in breast cancer.

### Transcription Factors Identification

Cistrome DB Toolkit database^6^ (Zheng et al., 2019) is a website tool that allows users to query transcription factors (TFs) that might regulate genes. We used the Cistrome DB Toolkit to predict which TFs are most likely to increase SAE1 expression in breast cancer. The correlation coefficient *R* > 0.3 is considered relevant.

### Gene Set Variation Analysis

Gene set variation analysis (GSVA) provides increased power to detect subtle pathway activity changes over a sample population in comparison with corresponding methods. In the study, the pathway activity of protein SUMOylation in TNBC were analyzed using R package “GSVA.”

### Statistical Analysis

In the study, *t*-test was applied to calculate the statistical correlation. Correlations between SAE1 expression and clinicopathological characteristics were performed by the chi-squared test. DFS and OS were calculated by the Kaplan-Meier method, and survival curves were compared *via* log-rank test. We conducted Cox regression analysis for the univariate and multivariate survival analyses. All *P*-values < 0.05 were considered statistically significant.

## Results

### The Level of SUMO1-Activating Enzyme Subunit 1 mRNA Was Highly Expressed in Triple-Negative Breast Cancer and Predicted Poor Prognosis

**FIGURE 1:**
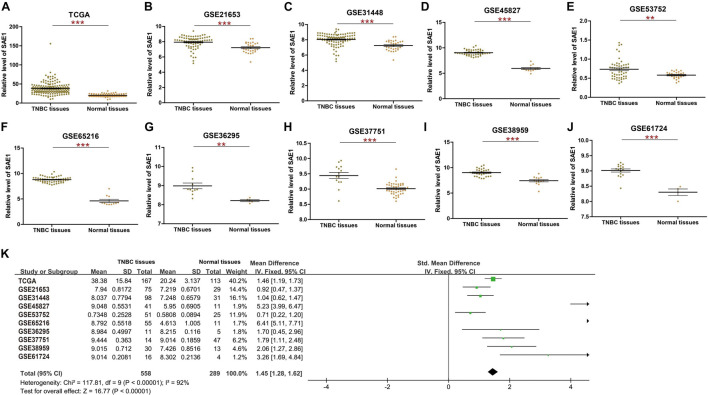
The mRNA expression of SAE1 in TNBC. **(A)** The mRNA expression of SAE1 for TNBC patients in TCGA database. **(B–I)** The mRNA expression of SAE1 for TNBC patients in GSE21653 **(B)**, GSE31448 **(C)**, GSE45827 **(D)**, GSE53752 **(E)**, GSE65216 **(F)**, GSE36295 **(G)**, GSE37751 **(H)**, GSE38959 **(I)**, and GSE6172 **(J)**. Meta-analysis of SAE1 expression in TNBC patients **(K)**. **p < 0.01; ***p < 0.001.

To examine the mRNA expression pattern of SAE1 in TNBC, publicly available datasets from TCGA and nine related GEO databases, including GSE21653, GSE31448, GSE45827, GSE53752, GSE65216, GSE36295, GSE37751, GSE38959, and GSE61724, were applied (Figures 1A–J). The mRNA level of SAE1 was found to be significantly increased in TNBC tissues (TCGA, *p* < 0.001; GSE21653, *p* < 0.001; GSE31448, *p* < 0.001; GSE45827, *p* < 0.001; GSE53752, *p* < 0.01; GSE65216, *p* < 0.001; GSE36295, *p* < 0.01; GSE37751, *p* < 0.001; GSE38959, *p* < 0.001; and GSE61724, *p* < 0.001). Moreover, meta-analysis containing 847 tissues from 10 TNBC databases mentioned above further demonstrated that the level of SAE1 mRNA was increased in TNBC (*p* < 0.001; Figure 1K). Next, we found that the average mRNA expression of SAE1 was higher in TNBC patients compared with luminal A or luminal B breast cancer subtypes based on GEO database GSE31448, GSE45827, and GSE65216 (Supplementary Figure 1). However, no significant differences were detected between TNBC- and HER2-enriched subtypes. These results could indicate that the increased expression of SAE1 might be more associated with ER-negative and PR-negative status.

**FIGURE 2:**
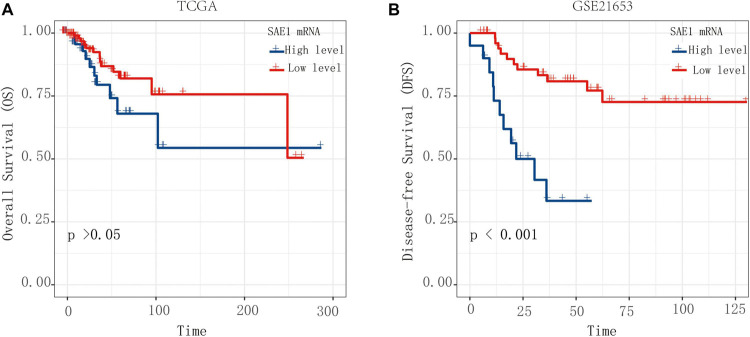
The OS and DFS of SAE1 for TNBC patients. The prognostic value of SAE1 mRNA expression for OS **(A)** and DFS **(B)** in TNBC patients by Kaplan-Meier analysis. The log-rank test was performed to evaluate survival difference with the best cutoff value.

In order to explore whether the mRNA expression of SAE1 could predict poor survival for TNBC patients, Kaplan-Meier survival analysis was performed for the OS and DFS. TNBC patients with high SAE1 expression had a significantly detrimental prognosis compared with those with low SAE1 expression (OS, *p* > 0.05; DFS, *p* < 0.001) (Figures 2A,B). Meanwhile, we performed GSVA to conduct GO analysis and assign protein SUMOlyation pathway activity estimates to individual samples and found that the activity of SUMOylation pathway was higher in TNBC patients compared with all other breast cancer subtypes (Supplementary Figure 2). The result of survival analysis indicated that high activity of SUMOylation pathway had a significantly detrimental DFS prognosis compared with those with low activity of SUMOylation pathway (Supplementary Figure 3).

### SUMO1-Activating Enzyme Subunit 1 mRNA Expression Is Positively Correlated With Forkhead Box M1 and Negatively Correlated With Methylation Site (cg14042711)

**FIGURE 3:**
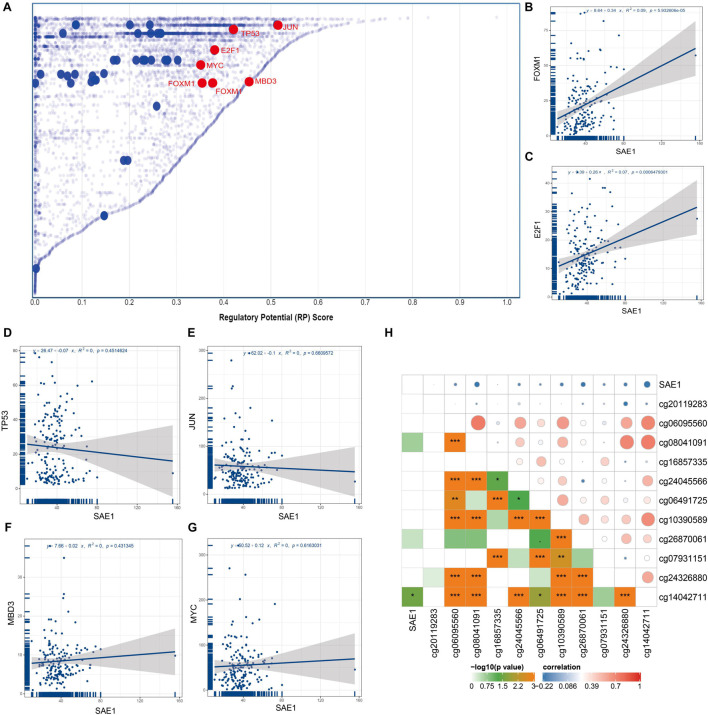
Relationships of SAE1 with TFs and DNA methylation. **(A)** TFs with high regulatory potential in MDA-MB-231 cell lines (10k distance to TSS). **(B)** Correlation between SAE1 and FOXM1 mRNA expression. **(C)** Correlation between SAE1 and E2F1 mRNA expression. **(D)** Correlation between SAE1 and TP53 mRNA expression. **(E)** Correlation between SAE1 and JUN mRNA expression. **(F)** Correlation between SAE1 and MBD3 mRNA expression. **(G)** Correlation between SAE1 and MYC mRNA expression. **(H)** Relationships of SAE1 with DNA methylation in TNBC. *p < 0.05; **p < 0.01; ***p < 0.001.

To identify members of a putative molecular network that might affect SAE1 expression, we examined TFs that might regulate SAE1 gene transcription using the Cistrome DB Toolkit. We checked the TNBC cell line MDA-MB-231 and found that jun proto-oncogene (JUN), tumor protein p53 (TP53), methyl-CpG-binding domain protein 3 (MBD3), E2F transcription factor 1 (E2F1), v-myc avian myelocytomatosis viral oncogene homolog (MYC), and forkhead box M1 (FOXM1) possessed the regulatory potential in MDA-MB-231 cell (Figure 3A). Subsequent analyses revealed that SAE1 mRNA and FOXM1 mRNA expression were positively correlated in TNBC tissues using TCGA database (Figure 3B, *r* = 0.34, *p* < 0.001). However, the expression of SAE1 mRNA was not significantly correlated with JUN, TP53, MBD3, E2F1, and MYC (Figures 3C–G). The transcription factor FOXM1 coordinates the expression of cell cycle-related genes and plays a pivotal role in tumorigenesis and cancer progression. To understand the role of FOXM1 in breast cancer, gene expression data (GSE55204) from breast cancer cells before and after FOXM1 knockdown to highlight specific FOXM1 transcriptional networks were performed by Bergamaschi et al. (2014). Based on the GSE55204 database, we found that when FOXM1 was knocked down (Supplementary Figure 4A), SAE1 expression decreased (Supplementary Figure 4B). Meanwhile, the expression of FOXM1 and SAE1 was positively correlated (Supplementary Figure 4C). These experimental results further support our conclusion.

The methylation of promoter is one of the critical mechanisms regulating gene expression which has been shown to control transcription in mammals. Previous studies have revealed that increased expressions of many genes were associated with hypomethylation of promoter. The association between the SAE1 expression and methylation of SAE1 for TNBC patient was detected *via* UCSC Xena. The associations between methylation of 11 SAE1 CpG sites and SAE1 expression are displayed in Figure 3H. The Pearson’s correlation coefficient between methylation site (cg14042711) and SAE1 is −0.32 (*p* < 0.05). However, we did not detect any significant correlation between 10 other SAE1 CpG sites and SAE1 expression. These results indicate that TFs and DNA methylation modifications might play a considerable role in TNBC processes by regulating SAE1 expression.

### SUMO1-Activating Enzyme Subunit 1 Expression Was Not Correlated With Immune Factors

**FIGURE 4:**
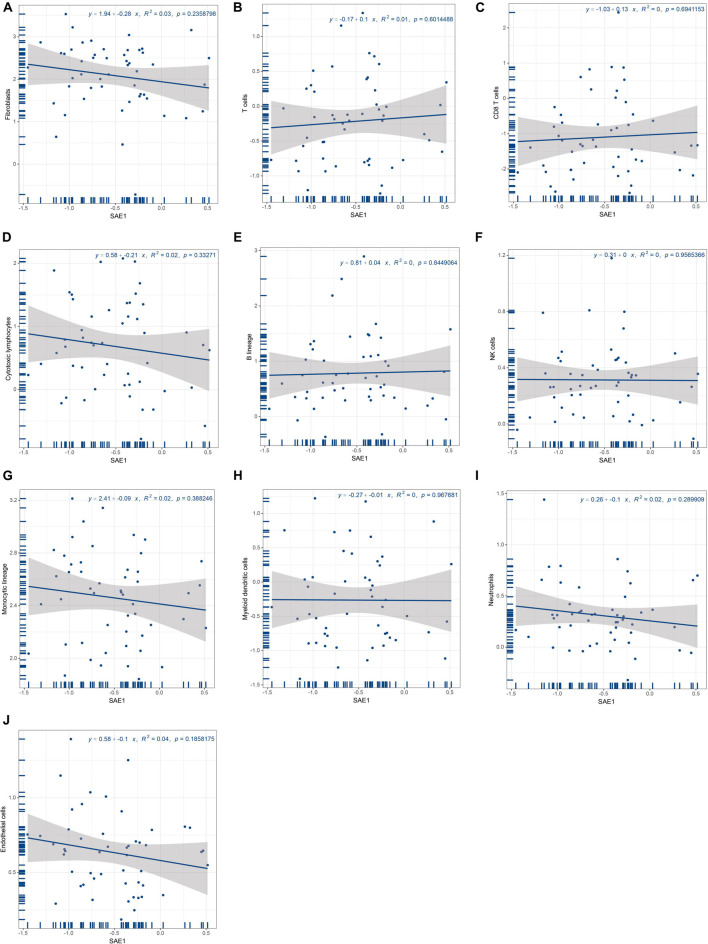
Relationships of SAE1 with tumor-infiltrating immune cells. **(A)** Correlation between SAE1 expression and fibroblasts. **(B)** Correlation between SAE1 expression and T cells. **(C)** Correlation between SAE1 expression and CD8 T cells. **(D)** Correlation between SAE1 expression and cytotoxic lymphocytes. **(E)** Correlation between SAE1 expression and B lineage. **(F)** Correlation between SAE1 expression and NK cells. **(G)** Correlation between SAE1 expression and monocytic lineage. **(H)** Correlation between SAE1 expression and myeloid dendritic cells. **(I)** Correlation between SAE1 expression and neutrophils. **(J)** Correlation between SAE1 expression and endothelial cells.

It is well-known that tumor-infiltrating immune cells could play an important role during cancer development and are of prognostic value in TNBC patients. Therefore, we performed a correlation analysis of SAE1 expression with the quantity of 10 tumor-infiltrating immune cells (T cells, CD8 T cells, cytotoxic lymphocytes, B lineage, NK cells, monocytic lineage, myeloid dendritic cells, neutrophils, endothelial cells, and fibroblasts). However, it is indicated that the expression of SAE1 was not correlated with different immune cell infiltrates (Figures 4A–J).

### The Genes Coexpressed With SAE1 Enriched in the Cell Cycle, DNA Repair, DNA Damage, and Proliferation

**FIGURE 5:**
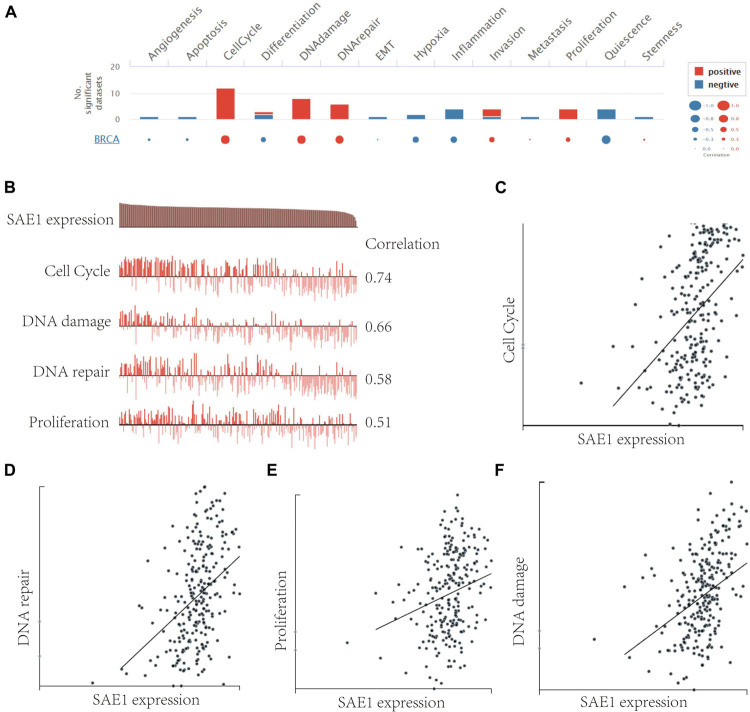
The function of SAE1 in breast cancer cells. **(A)** Single-cell analysis displayed the correlation between SAE1 and 14 functional states (including angiogenesis, apoptosis, invasion, EMT, differentiation, proliferation, DNA damage, metastasis, hypoxia, inflammation, cell cycle, DNA repair, stemness, and quiescence). **(B)** Single-cell analysis indicated that SAE1 is primarily involved in regulation of the cell cycle, DNA damage and repair, and proliferation in breast cancer. **(C)** Correlation between SAE1 expression and cell cycle. **(D)** Correlation between SAE1 expression and DNA repair. **(E)** Correlation between SAE1 expression and cell proliferation. **(F)** Correlation between SAE1 expression and DNA damage.

To better understand the relevance and underlying mechanisms of SAE1 expression in breast cancer, we investigated the functional state of SAE1 in single-cell level *via* the CancerSEA database. Figure 5A displays the correlation between SAE1 and 14 functional states (including angiogenesis, apoptosis, invasion, EMT, differentiation, proliferation, DNA damage, metastasis, hypoxia, inflammation, cell cycle, DNA repair, stemness, and quiescence). Results indicated that SAE1 was positively correlated with cell cycle, DNA repair, DNA damage, and proliferation (Figures 5B–F).

**FIGURE 6:**
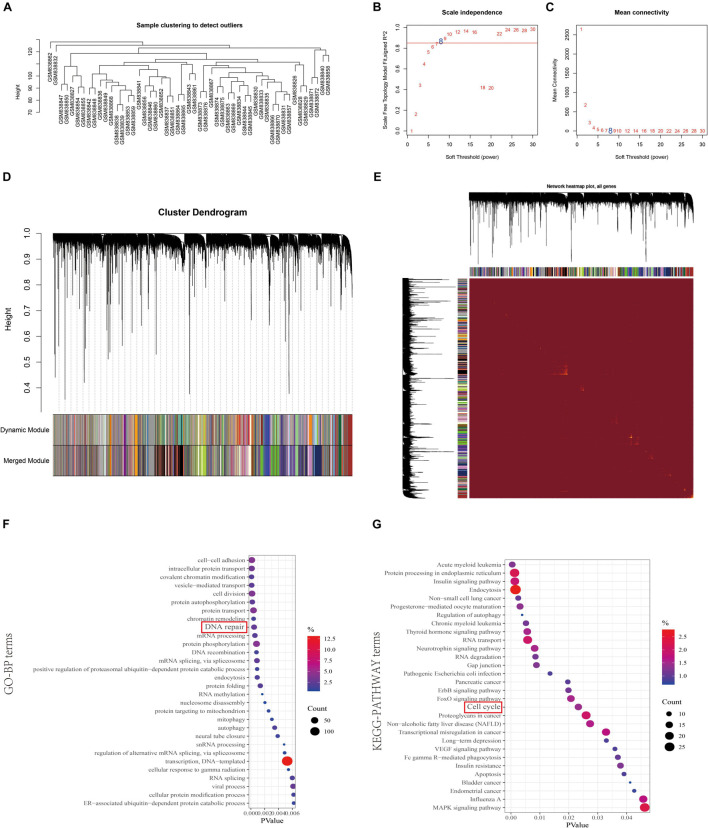
Identification of coexpression module genes associated with SAE1 using the WGCNA. **(A)** Clustering dendrogram of TNBC tissues. **(B)** Relationship between scale-free topology model fit and soft thresholds (powers). **(C)** Relationship between the mean connectivity and various soft thresholds. **(D)** Dendrogram of modules identified by WGCNA. **(E)** Topological overlap matrix among detected genes from RNAseq. **(F,G)** GO-BP **(F)** and KEGG pathway **(G)** network for the target genes in green model.

To reveal the highly correlated genes and coexpression networks of SAE1 gene in TNBC patients, WGCNA analysis was conducted. Fifty-one TNBC samples obtained from GSE53752 database was used to build the WGCNA network (Figures 6A–E). We calculated the network topology for soft-thresholding powers from 1 to 30 to choose the best threshold. One of the most critical parameters in WGCNA network construction is the power value, which affects the average connectivity and independence of each coexpression module. Power value 8 was the lowest power for the scale-free topology. The coexpression similarity matrix was transformed into adjacency matrix, and the topological overlap matrix (TOM) was calculated. The dynamic tree cut analysis gave rise to 46 modules with different colors, among which the genes coexpressed with SAE1 belong to the green module containing 1,176 genes.

In order to gain further insights into the function of 1,176 genes in green module, we used DAVID database to analyze GO and KEGG pathway (Figures 6F,G). The terms of biological processes (BP) were cell-cell adhesion, intracellular protein transport, covalent chromatin modification, vesicle-mediated transport, cell division, protein autophosphorylation, protein transport, chromatin remodeling, DNA repair, mRNA processing, and protein phosphorylation. The terms of KEGG pathway terms were acute myeloid leukemia, protein processing in endoplasmic reticulum, insulin signaling pathway, endocytosis, non-small cell lung cancer, progesterone-mediated oocyte maturation, regulation of autophagy, chronic myeloid leukemia, thyroid hormone signaling pathway, and cell cycle.

**FIGURE 7:**
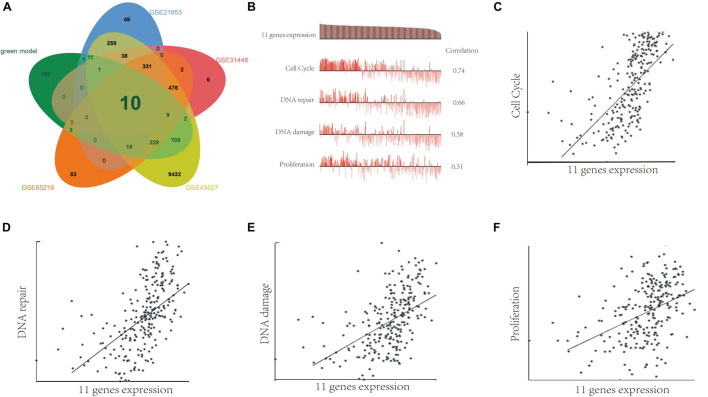
The function of SAE1 coexpression genes in breast cancer cells. **(A)** Venn diagrams showing relationships between DEGs and green model genes. **(B)** Single-cell analysis indicated that those 11 genes are primarily involved in regulation of the cell cycle, DNA damage and repair, and proliferation in breast cancer. **(C)** Correlation between 11 gene expression and cell cycle. **(D)** Correlation between 11 gene expression and DNA repair. **(E)** Correlation between SAE1 expression and DNA damage. **(F)** Correlation between 11 gene expression and cell proliferation.

Meanwhile, we analyzed the differentially expressed genes (DEGs) between TNBC tissues and adjacent normal tissues by using GSE45827, GSE65216, GSE31448, and GSE21653 databases. Figure 7A shows that the 1,176 genes in green module and DEGs in GSE45827, GSE65216, GSE31448, and GSE21653 have 10 overlap genes including minichromosome maintenance complex component 5 (MCM5), polo-like kinase 1 (PLK1), PHD finger protein 19 (PHF19), ubiquitin-conjugating enzyme E2S (UBE2S), secretion associated, Ras related GTPase 1A (SAR1A), extra spindle pole bodies homolog 1 (ESPL1), coactosin-like F-actin binding protein 1 (COTL1), antisilencing function 1B histone chaperone (ASF1B), apolipoprotein O (APOO), and transcription factor 19 (TCF19). Next, single-cell analysis was performed to investigate the functions of the 11 genes including the 10 overlap genes and SAE1 gene in breast cancer *via* CancerSEA website. Results indicated that these 11 genes were positively correlated with cell cycle, DNA repair, DNA damage, and proliferation (Figures 7B–F).

### Construction of a Prognostic Scoring Model Based on SUMO1-Activating Enzyme Subunit 1 and Polo Like Kinase 1 Expression

**FIGURE 8:**
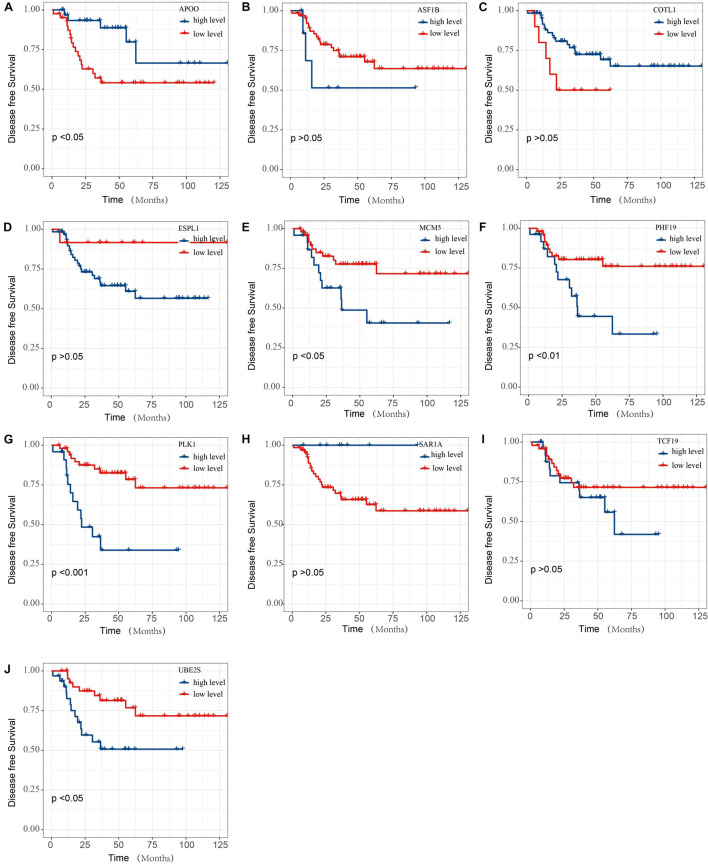
The DFS of SAE1 coexpression genes for TNBC patients. The prognostic value of **(A)** APOO, **(B)** ASF1B, **(C)** COTL1, **(D)** ESPL1, **(E)** MCM5, **(F)** PHF19, **(G)** PLK1, **(H)** SAR1A, **(I)** TCF19, and **(J)** UBE2S mRNA expression for DFS in TNBC patients by Kaplan-Meier analysis. The log-rank test was performed to evaluate survival difference with the best cutoff value.

The prognostic analysis of the 10 overlap genes for TNBC patients were further evaluated (Figure 8). TNBC patients with low APOO expression exhibited significantly decreased DFS. TNBC patients with low MCM5, PHF19, PLK1, and UBE2S expression exhibited significantly increased DFS. However, there was not any significant relationship between the expression of ASF1B, COTL1, ESPL1, SAR1A, and TCF19 expression and the DFS of TNBC patients.

**FIGURE 9:**
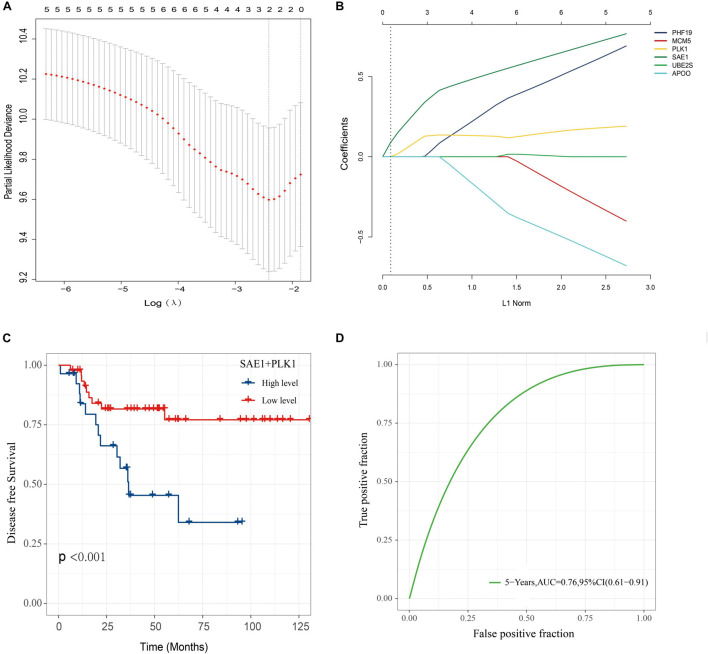
Construction of a classifier to predict disease-free survival in TNBC patients. **(A)** Partial likelihood deviance of DFS for the LASSO coefficient profiles. **(B)** LASSO coefficient profiles of the SAE1, APOO, MCM5, PHF19, PLK1, and UBE2S expression for DFS. **(C)** Kaplan-Meier survival analysis showing the impact of the classifier on DFS. **(D)** ROC curves compare the prognostic accuracy of the classifier in TNBC patients using AUCs at 5 years to assess prognostic accuracy.

In order to construct a risk score model for predicting DFS of TNBC, LASSO Cox regression model was used to build a prognostic classifier, which included SAE1, APOO, MCM5, PHF19, PLK1, and UBE2S (Figures 9A,B). Using the LASSO Cox regression models, we calculated a risk score for each patient based on those six gene expression: risk score = (PLK1 × 0.1122) + (SAE1 × 0.3116). Survival analysis revealed that the survival time of TNBC patients in the high-risk group was significantly shorter than those patients in the low-risk group (Figure 9C). Then, the model reliability was verified through the ROC curves, and the results exhibited that the AUC value was 0.76 (Figure 9D). The above results indicated that the SAE1 and PLK1 risk assessment model had predictive value for the prognosis of TNBC patients.

### The Level of SUMO1-Activating Enzyme Subunit 1 Protein Was Highly Expressed in Triple-Negative Breast Cancer and Predicted Poor Prognosis Through Immunohistochemical

**FIGURE 10:**
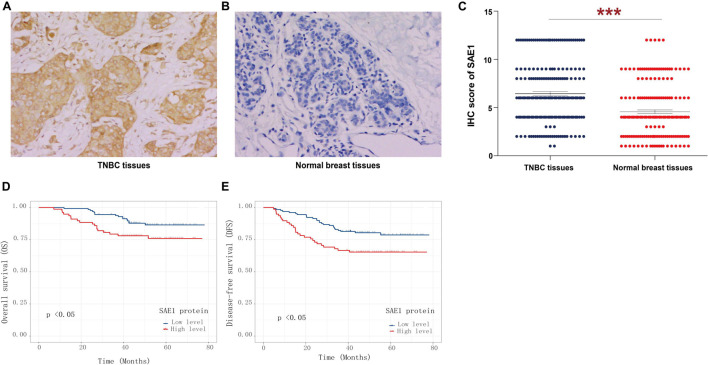
Immunohistochemical (IHC) analyses of SAE1 protein expression in TNBC. Representative IHC images of SAE1 protein expression in TNBC tissues **(A)** and normal breast tissues **(B)**. **(C)** Histogram of IHC score for SAE1 in TNBC patients. **(D,E)** The prognostic value of SAE1 protein expression for OS **(D)** and DFS **(E)** in TNBC patients by Kaplan-Meier analysis. ***p < 0.001.

Finally, the protein expression of SAE1 in TNBC was detected *via* IHC staining analysis. SAE1 protein expression was demonstrated significantly increased in 207 TNBC tissues compared with the paired adjacent normal breast tissues (Figures 10A–C). In our study, we found that SAE1 mainly localized to the cytoplasm but not the nucleus in TNBC patients (Supplementary Figure 5). The clinicopathological characteristics of 207 TNBC patients are presented in Table 1, and the correlations between relevant clinicopathological factors and SAE1 protein expression were analyzed. However, no associations between clinicopathological features and SAE1 expression were observed.

**TABLE 1:** Characteristics of triple-negative breast cancer patients and their SAE1 expression level.

Characteristics	All patients		Low SAE1	High SAE1	*P*-value^a^
	*n* = 207		*n* = 129	*n* = 78	
	No.	(%)	No.	No.	
Age at diagnosis (years)					0.990
≤50	93	44.9	58	35	
>50	114	55.1	71	43	
Tumor size					0.450
≤2 cm	86	41.5	51	35	
>2 cm	121	58.5	78	43	
Lymph node metastasis					0.338
No	124	59.9	74	50	
Yes	83	40.1	55	28	
Tumor grade					0.770
I + II	77	37.2	47	30	
III	130	62.8	82	48	
Lymphovascular invasion					0.452
Yes	134	64.7	81	53	
No	73	35.3	48	25	
Type of surgery					0.364
Total mastectomy	195	94.2	123	72	
Breast conserving surgery	12	5.8	6	6	

*^a^The *P*-value was calculated among all groups by the Chi-square test.*

Survival analysis was also applied to analyze the relationship between SAE1 protein expression and the prognosis of these 207 TNBC patients. The results of Kaplan-Meier curve implied that TNBC patients with lower protein expression of SAE1 have better prognosis in both OS and DFS than those with higher expression (Figures 10D,E). Meanwhile, univariate and multivariate Cox analyses were performed to identify the independent prognostic factor for poor survival in TNBC patients (Tables 2, 3). Lymph node metastasis, tumor grade, LVI, and SAE1 expression were determined to be independent prognostic factors for OS of TNBC patients by multivariate Cox analysis. For DFS, tumor size, lymph node metastasis, tumor grade, LVI, and SAE1 expression were identified to be independent prognostic factors in TNBC patients.

**TABLE 2:** Univariate Cox proportional hazard model for disease-free survival (DFS) and overall survival (OS) in TNBC patients with adjuvant chemotherapy.

Variables	DFS		OS	
	HR (95% CI)	*P*-value^a^	HR (95% CI)	*P*-value^a^
**Age (years)**				
≤50	Reference		Reference	
>50	0.55 (0.32–0.96)	0.034	0.91 (0.47–1.79)	0.793
**Tumor size**				
≤2 cm	Reference		Reference	
>2 cm	1.99 (1.09–3.61)	0.024	1.84 (0.88–3.84)	0.106
**Lymph nodes metastasis**				
No	Reference		Reference	
Yes	2.30 (1.33–3.98)	0.003	2.55 (1.27–5.09)	0.008
**Grade**				
I + II	Reference		Reference	
III	2.25 (1.18–4.27)	0.014	2.49 (1.09–5.73)	0.031
**Lymphovascular invasion**				
No	Reference		Reference	
Yes	1.87 (1.09–3.20)	0.023	1.78 (0.91–3.50)	0.094
**Type of surgery**				
Total mastectomy	Reference		Reference	
Breast conserving surgery	0.60 (0.15–2.47)	0.482	0.48 (0.07–3.48)	0.464
**SAE1 expression**				
Low	Reference		Reference	
High	1.98 (1.16–3.40)	0.013	2.06 (1.05–4.05)	0.035

*HR, hazard ratio; CI, confidence interval; DFS, disease-free survival; OS, overall survival.*

*^a^The *P*-value was adjusted by the univariate Cox proportional hazard regression model.*

**TABLE 3:** Multivariate Cox proportional hazard model for disease-free survival (DFS) and overall survival (OS) in TNBC patients with adjuvant chemotherapy.

Variables	DFS		OS	
	HR (95% CI)	*P*-value^a^	HR (95% CI)	*P*-value^a^
**Age (years)**				
≤50	Reference		N/A	
>50	0.74 (0.42–1.30)	0.297		
**Tumor size**				
≤2 cm	Reference		N/A	
>2 cm	1.90 (1.04–3.50)	0.038		
**Lymph nodes metastasis**				
No	Reference		Reference	
Yes	1.90 (1.06–3.41)	0.031	2.48 (1.22–5.04)	0.012
**Grade**				
I + II	Reference		Reference	
III	2.20 (1.15–4.19)	0.017	2.46 (1.07–5.66)	0.034
**Lymphovascular invasion**				
No	Reference		Reference	
Yes	1.81 (1.03–3.17)	0.039	1.59 (0.79–3.18)	0.193
**SAE1 expression**				
Low	Reference		Reference	
High	2.40 (1.39–4.15)	0.002	2.50 (1.26–4.98)	0.009

*HR, hazard ratio; CI, confidence interval; DFS, disease-free survival; OS, overall survival.*

*^a^The *P*-value was adjusted by the multivariate Cox proportional hazard regression model.*

## Discussion

SUMO1-activating enzyme subunit 1 is a component of SUMO which are small protein modifiers capable of regulating cellular localization and function of target proteins that act as linchpins in SUMOylation, a post-translational modification involved in cellular processes such as regulation of gene transcription, DNA repair, cell-cycle regulation, and apoptosis. Potential relevancy between malignant diseases and SUMOylation pathway has been put forward and analogously the aberrant SAE1 was proved to be a partaker in cancer development. Reported overexpression of SAE1 in a stage-dependent manner in glioma by activating AKT SUMOylation-mediated signaling pathways suggested a probable role of SAE1 in cancer initiation and progression. While the reported opposite tendency of prognostic influence on gastric cancer and hepatocellular carcinoma by differential SAE1 expression indicated the uncertain association between SAE1 and malignant disease.

To study the potential mechanism of malignancy that related to SAE1, SAE1 expression analysis and prognostic analysis by Kaplan-Meier method were operated to evaluate the preliminary clinical significance based on the publicly available TCGA and GEO databases. Compared with adjacent normal tissues, the mRNA expression of SAE1 was increased in TNBC tissues, and the upregulation of SAE1 in TNBC tumor tissues was revealed with a poor prognosis (DFS) for TNBC patients.

Forkhead box M1 is a key transcription factor that plays an important role in the development of embryos, the homeostasis of mature tissues, and the occurrence and progression of malignant tumors (Bella et al., 2014). It regulates many important physiological functions, including promoting the repair of DNA damage; regulating the cell cycle G1/S phase, G2/S phase transition, and M phase process to ensure the progress of cell mitosis. FOXM1 is upregulated in many malignant tumors (Kim et al., 2006). Tumor treatment methods usually include radiotherapy or drug treatment that can damage DNA, such as platinum compounds, anthracyclines, topoisomerase inhibitors, alkylating agents, etc., which are the main drugs for clinical treatment of tumors (Johnson and Seidman, 2005; Palmieri et al., 2010). However, most DNA-damaging drugs often develop drug resistance, which is the main reason for the failure of tumor treatment (Colak and Medema, 2014). Studies have found that FOXM1 plays a key role in the resistance of DNA damaging drugs. If it is activated abnormally or its expression is elevated, it may promote the development of resistance to DNA-damaging drugs (Myatt and Lam, 2007, 2008). Here, we confirmed that FOXM1 expression was positively correlated with SAE1 expression, suggesting that SAE1 may be involved in the resistance to DNA damaging drugs. TF inhibitors might also help to improve the treatments of TNBC patients.

TCGA database was quoted synchronously to determine whether the SAE1 level would be manipulated by adjusting DNA methylation in TNBC. The result presented a significant negative correlation between SAE1 expression and SAE1 methylation site (cg14042711) level. Simultaneously, correlation analysis between SAE1 and the quantity of tumor-infiltrating immune cells in the TNBC tumor microenvironment displayed a negative outcome.

To further examine the effects of elevated SAE1 levels, CancerSEA was performed for analysis. The results indicate that SAE1 may affect the progression of breast cancer by regulating the cell cycle, DNA damage, DNA repair, and cell proliferation. Next, we implemented the WGCNA for the determination of SAE1 correlated genes as well as the enrichment analysis for gene functional study. The result showed the SAE1 coexpression genes were enriched into the green module which contains 1,176 genes that mainly clustered in cell cycle and DNA repair. The 1,176 genes in green module and DEGs in GSE45827, GSE65216, GSE31448, and GSE21653 have 10 overlap genes. Results indicated that these 11 genes including SAE1 and 10 overlap genes were also positively correlated with cell cycle, DNA repair, DNA damage, and proliferation. This is consistent with contemporary knowledge that SUMOs such as SAE1 are essential for the regulation of several cellular processes, including transcriptional regulation, transcript processing, genomic replication, and DNA damage repair (Sacher et al., 2006; Hang et al., 2011; Psakhye and Jentsch, 2012; Sarangi et al., 2014; Schimmel et al., 2014; Sarangi and Zhao, 2015). The expressions of these 10 genes were further examined by survival analysis, and the results indicated that SAE1, APOO, MCM5, PHF19, PLK1, and UBE2S were significantly correlated with the prognosis. Meanwhile, we identified two core genes including SAE1 and PLK1 closely related to the DFS of TNBC and constructed a survival prediction model based on SAE1 and PLK1 expression. The AUC value of the ROC curves in this model was 0.76, which proved that the model was reliable.

Finally, our IHC findings suggested that the protein expression of SAE1 was upregulated in TNBC tissues of 207 patients. According to survival analysis, SAE1 protein was associated with both OS and DFS separately. These observations collectively provide a new idea for the prognosis of TNBC patients.

## Conclusion

To generalize, detection on expression patterns of SAE1 and its prognostic significance in TNBC was primarily implemented by bioinformatic analysis and IHC which showed a positive correlation between SAE1 downregulation and better prognosis. SAE1 expression correlated with DNA methylation and TF. It is therefore of great clinical importance to investigate DNA methyltransferase and TF inhibitors that might decrease the expression of SAE1.

## Data Availability Statement

The original contributions presented in the study are included in the article/Supplementary Material, further inquiries can be directed to the corresponding authors.

## Ethics Statement

Written informed consent was obtained from the individual(s) for the publication of any potentially identifiable images or data included in this article.

## Author Contributions

QW and WZ: conceptualization. LD: methodology. QL, YYL, and HXL: software. LX, LL, and YC: validation. JH and MJ: formal analysis. HX and JZ: data curation. QW and YXL: writing—original draft preparation. CS and YaL: writing—review and editing. All authors contributed to the article and approved the submitted version.

## Conflict of Interest

The authors declare that the research was conducted in the absence of any commercial or financial relationships that could be construed as a potential conflict of interest.

## Publisher’s Note

All claims expressed in this article are solely those of the authors and do not necessarily represent those of their affiliated organizations, or those of the publisher, the editors and the reviewers. Any product that may be evaluated in this article, or claim that may be made by its manufacturer, is not guaranteed or endorsed by the publisher.

## Footnotes

1
http://www.cbioportal.org/

2
http://www.ncbi.nlm.nih.gov/geo/

3
http://xena.ucsc.edu/

4
https://david.ncifcrf.gov/

5
http://biocc.hrbmu.edu.cn/CancerSEA/

6
http://dbtoolkit.cistrome.org

## Supplementary Material

Supplementary Figure 1 The mRNA expression of SAE1 in breast cancer. The mRNA expression of SAE1 in breast cancer patients based on GSE31448 **(A)**, GSE45827 **(B)**, and GSE65216 **(C)**. ns, *p* > 0.05; ^∗^*p* < 0.05; ^∗∗∗^*p* < 0.001.

Supplementary Figure 2 The pathway activity of SUMOylation in breast cancer. The pathway activity of SUMOylation in breast cancer based on GE21635. ns, *p* > 0.05; ^∗^*p* < 0.05.

Supplementary Figure 3 The OS and DFS of protein SUMOlyation pathway for TNBC patients. The prognostic value of protein SUMOlyation pathway for OS **(A)** and DFS **(B)** in TNBC patients by Kaplan-Meier analysis.

Supplementary Figure 4 Forkhead box M1 (FOXM1) regulates the expression of SAE1. The expression of FOXM1 **(A)** and SAE1 **(B)** in breast cancer cells transfected with control or FOXM1 siRNA base on GSE55204. Correlation between SAE1 expression and FOXM1 expression.

Supplementary Figure 5 Forkhead box M1 (FOXM1) regulates the expression of SAE1. Another two representative IHC images of SAE1 protein expression in TNBC tissues.
