# Supplementary figures and images for: The Expression and Prognostic Value of SUMO1-Activating Enzyme Subunit 1 and Its Potential Mechanism in Triple-Negative Breast Cancer
Source: Front Cell Dev Biol. 2021 Sep 21;9:729211. doi: 10.3389/fcell.2021.729211 (PMC8490707; doi:10.3389/fcell.2021.729211)

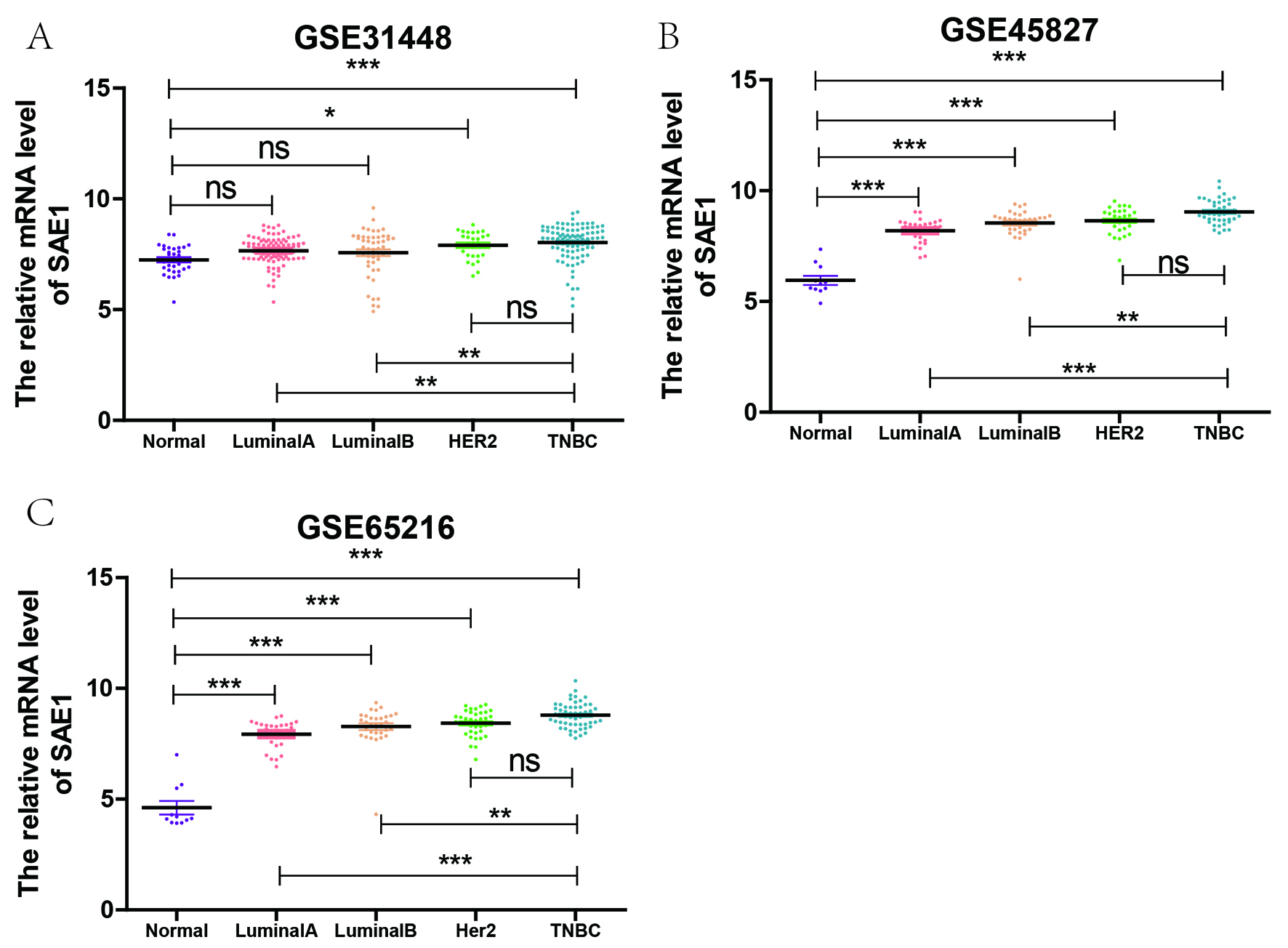

Supplement: Supplementary Figure 1 — The mRNA expression of SAE1 in breast cancer. The mRNA expression of SAE1 in breast cancer patients based on GSE31448 (A), GSE45827 (B), and GSE65216 (C). ns, p > 0.05; ∗p < 0.05; ∗∗∗p < 0.001. [file Image_1.JPEG]

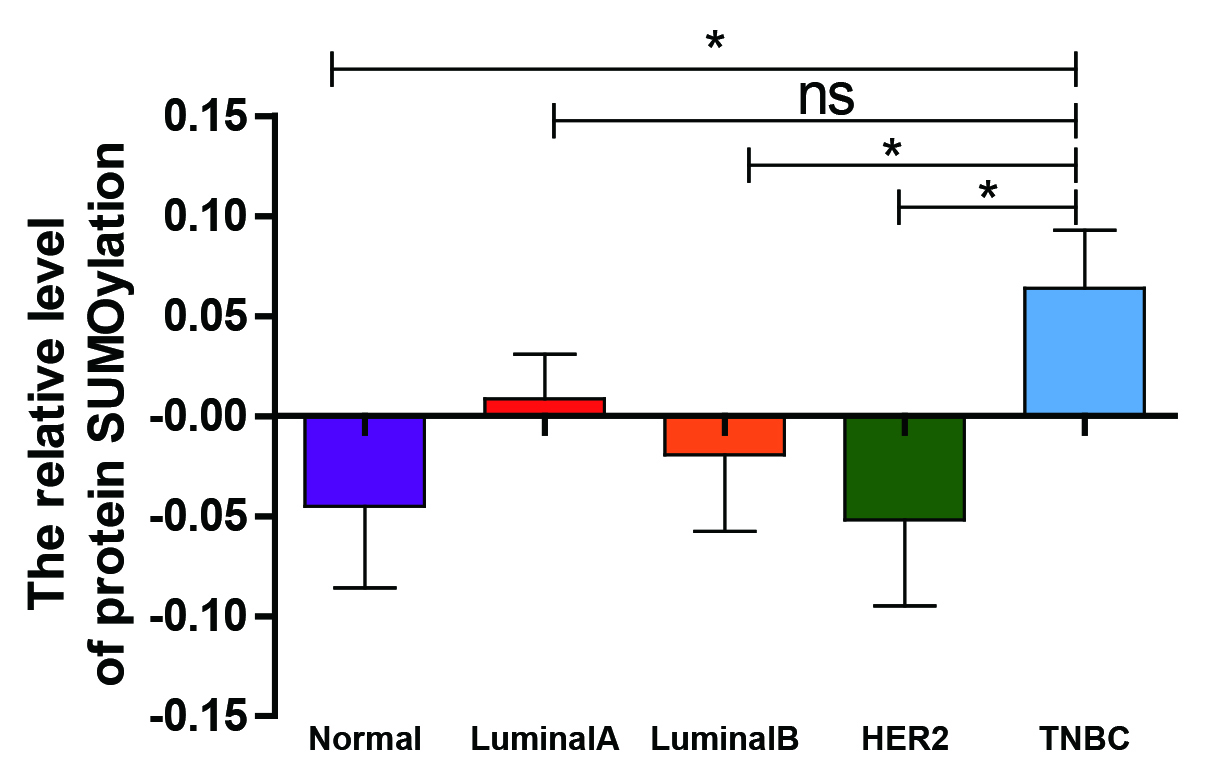

Supplement: Supplementary Figure 2 — The pathway activity of SUMOylation in breast cancer. The pathway activity of SUMOylation in breast cancer based on GE21635. ns, p > 0.05; ∗p < 0.05. [file Image_2.JPEG]

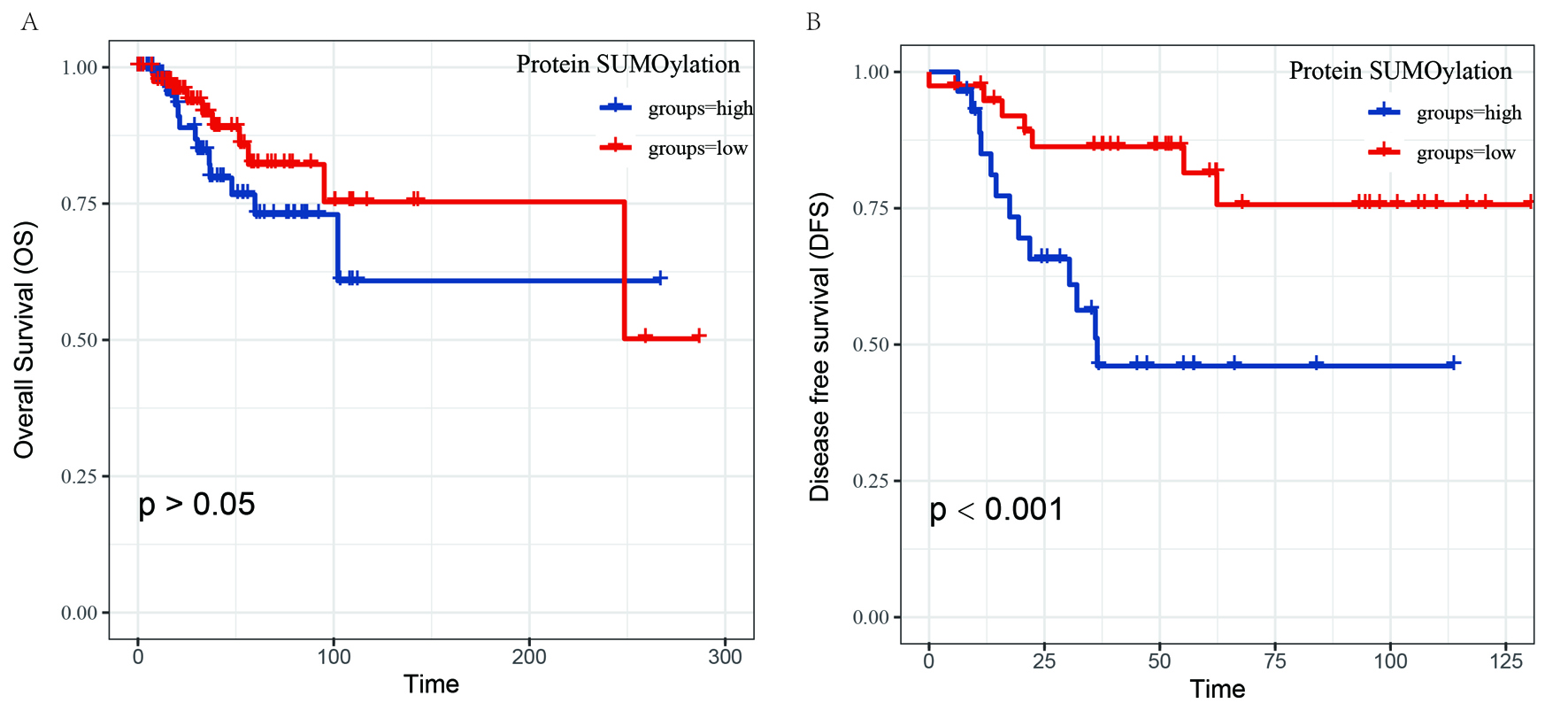

Supplement: Supplementary Figure 3 — The OS and DFS of protein SUMOlyation pathway for TNBC patients. The prognostic value of protein SUMOlyation pathway for OS (A) and DFS (B) in TNBC patients by Kaplan-Meier analysis. [file Image_3.JPEG]

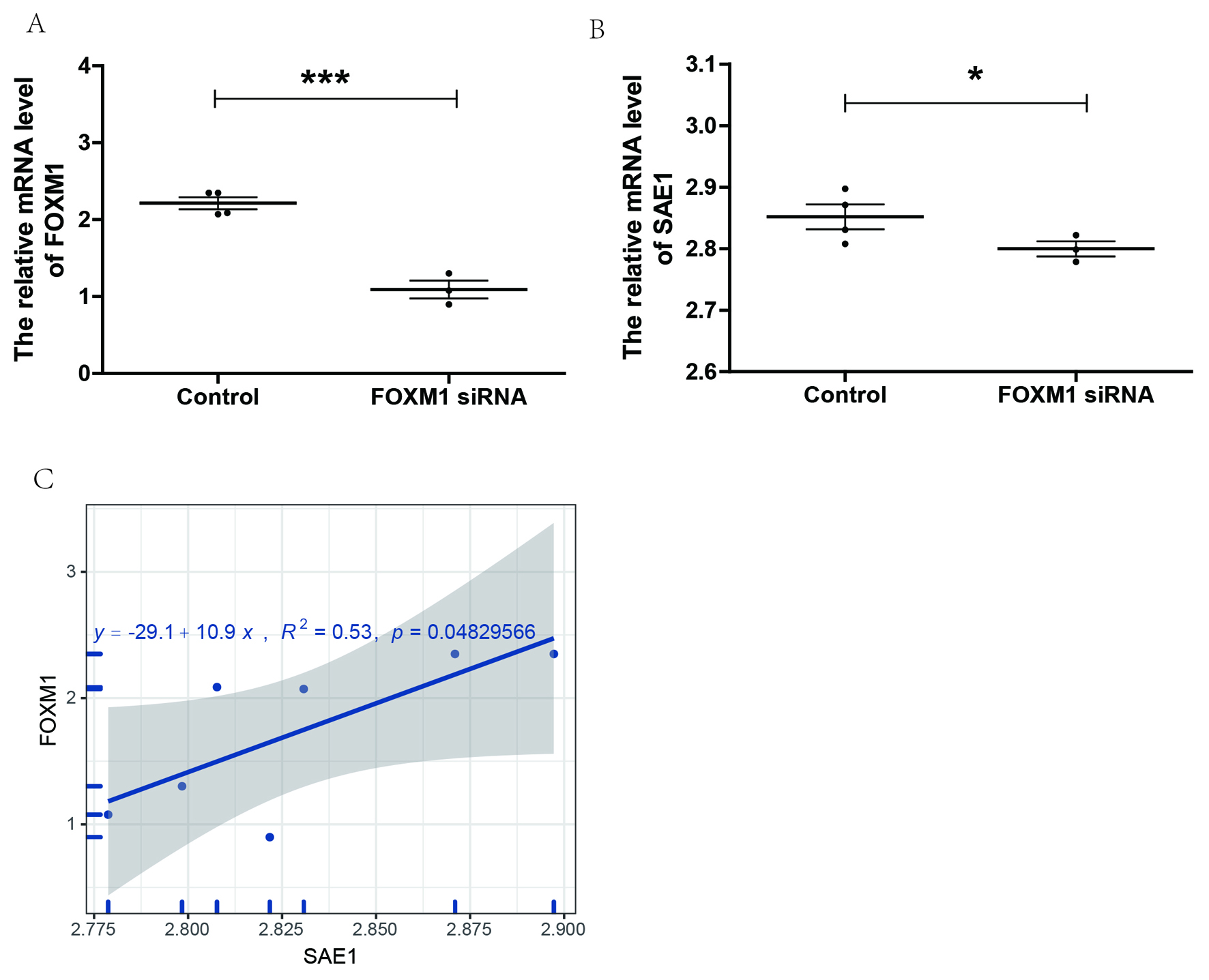

Supplement: Supplementary Figure 4 — Forkhead box M1 (FOXM1) regulates the expression of SAE1. The expression of FOXM1 (A) and SAE1 (B) in breast cancer cells transfected with control or FOXM1 siRNA base on GSE55204. Correlation between SAE1 expression and FOXM1 expression. [file Image_4.JPEG]

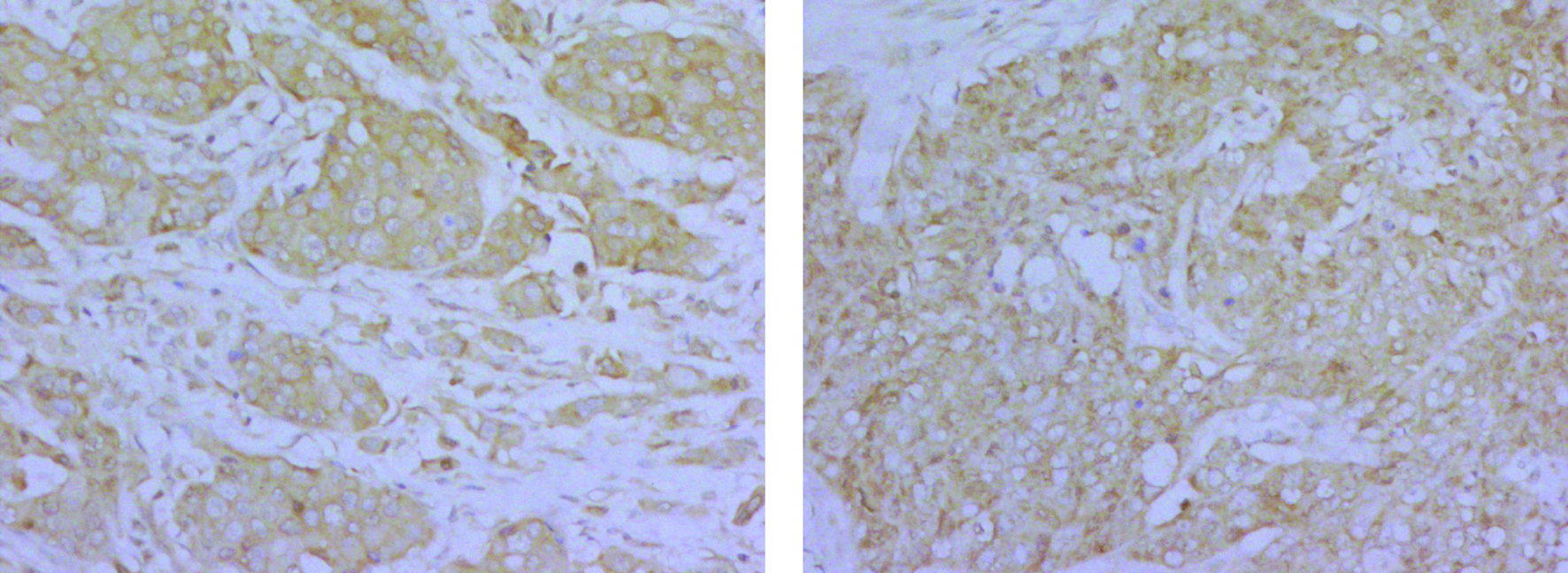

Supplement: Supplementary Figure 5 — Forkhead box M1 (FOXM1) regulates the expression of SAE1. Another two representative IHC images of SAE1 protein expression in TNBC tissues. [file Image_5.JPEG]
